# Papillo-Choledochectomy in the Operative Management of Mucosal Neoplasms of the Periampullary Region

**DOI:** 10.1155/1993/43212

**Published:** 1993

**Authors:** Kogoro Kasahara, Ken Saito, Yasuo Kondo, Toshihiko Yasuda, Yoshikazu Yasuda, Masatoshi Nakada, Hideo Nagai, Kyotaro Kanazawa

**Affiliations:** Departments of Surgery and Pathology Jichi Medical School Minami-kawachimachi Tochigi 329–04 Japan

## Abstract

Two patients with mucosal cancer of the periampullary region were treated with papillocholedochectomy, which entails removal of the papilla of Vater and the whole length of the common bile duct. The neoplasm is dissected out through the plane between the duodenal circular and longitudinal muscles, deep to the sphincter of Oddi and the fibromuscular layer of the bile duct. Pathological examination showed that cancer was confined to the mucosal layer without stromal invasion, and that the operation achieved radical cure. For mucosal cancer, papillo-choledochectomy is an alternative to pancreatoduodenectomy, provided that repeated frozen-section studies confirm the completeness of excision.


*HPB Surgery*, 1993, Vol. 6, pp. 211–217
Reprints available directly from the publisher
Photocopying permitted by license only

© 1993 Harwood Academic Publishers GmbH
Printed in the United States of America

### CASE REPORT

### PAPILLO-CHOLEDOCHECTOMY IN THE OPERATIVE MANAGEMENT OF MUCOSAL NEOPLASMS OF THE PERIAMPULLARY REGION

KOGORO KASAHARA, KEN SAITO, YASUO KONDO, TOSHIHIKO YASUDA, YOSHIKAZU YASUDA, MASATOSHI NAKADA, HIDEO NAGAI and KYOTARO KANAZAWA

*Departments of Surgery and Pathology, Jichi Medical School, Minami-kawachimachi, Tochigi, 329–04, Japan*

*(Received 28 January 1992)*

Two patients with mucosal cancer of the periampullary region were treated with papillo-choledochectomy, which entails removal of the papilla of Vater and the whole length of the common bile duct. The neoplasm is dissected out through the plane between the duodenal circular and longitudinal muscles, deep to the sphincter of Oddi and the fibromuscular layer of the bile duct. Pathological examination showed that cancer was confined to the mucosal layer without stromal invasion, and that the operation achieved radical cure. For mucosal cancer, papillo-choledochectomy is an alternative to pancreatoduodenectomy, provided that repeated frozen-section studies confirm the completeness of excision.

KEY WORDS: Papillo-choledochectomy, periampullary mucosal cancer

### INTRODUCTION

Tiny neoplasms are increasingly encountered in the periampullary region[1–7>]. With superficial biopsy, either endoscopic or operative, it can be difficult to differentiate between adenoma, mucosal carcinoma and invasive carcinoma. If the lesion is misdiagnosed as a benign adenoma, local excision can be followed by tumour recurrence[4,8]. On the other hand, a mucosal cancer wrongly diagnosed as an invasive cancer may lead to an appropriately radical operation, namely pancreatoduodenectomy[7,9,10].

Small mucosal neoplasms, whether adenomas or even mucosal carcinomas, can theoretically be cured by local excision, though a wide surgical margin is desirable to allow for understaging on biopsy. We describe two patients with mucosal cancer of the periampullary region who were treated with a new operative procedure, papillo-choledochectomy, which incorporates a wide surgical margin. The operation entails removal of the papilla of Vater and common bile duct followed by cholecystectomy, hepaticojejunostomy, and reconstruction of the main pancreatic duct.

Address correspondence to: Kogoro Kasahara, M.D., Department of Surgery, Jichi Medical School, Minami-kawachimachi, Tochigi, 329–04, Japan

### CASE REPORTS

### *Case 1*

A 63-year-old man was admitted with right hypochondrial pain and deranged liver function tests. Ultrasonography showed an enlarged gallbladder containing stones and a dilated extrahepatic bile duct. At endoscopy the papilla of Vater was swollen, and a neoplastic lesion was suspected; papillotomy was performed. A biopsy specimen disclosed tubulovillous adenoma with moderate atypia. Following transfer to our unit, repeat duodenoscopy showed a flat, elevated tumour with a granular surface replacing the papilla of Vater; further biopsies confirmed tubular adenoma.

At laparotomy, a sesile tumour (2.2 × 1.8 cm) was exposed through a duodenotomy. The common bile duct orifice was free of tumour proximally but involved distally. The pancreatic duct orifice opened separately and was completely surrounded by tumour. The tumour was excised together with the major papilla and a 3 mm disc of surrounding normal mucosa. Excision was carried out through the surgical plane between the circular and longitudinal muscles of the duodenum. Frozen section showed a non-invasive mucosal neoplasm involving the bile duct not the main pancreatic duct. As the tumour extended to the proximal margin of the bile duct, an additional 3–4 mm of bile duct was removed, but frozen section still showed involvement of the margin. Cholecystectomy was performed, and the common bile duct (including the cystic duct confluence) was separated from the surrounding pancreatic tissue, and resected completely right down to the duodenal lumen (Figure 1). The biliary tree was reconstructed by Roux-en-Y hepaticojejunostomy. The duodenal muscular and mucosal defects were primarily closed with reconstruction of the pancreatic ductal opening. Recovery was uncomplicated, and the patient remains well endoscopically free of disease at 31 months. The final histological diagnosis was of well differentiated papillotubular adenocarcinoma

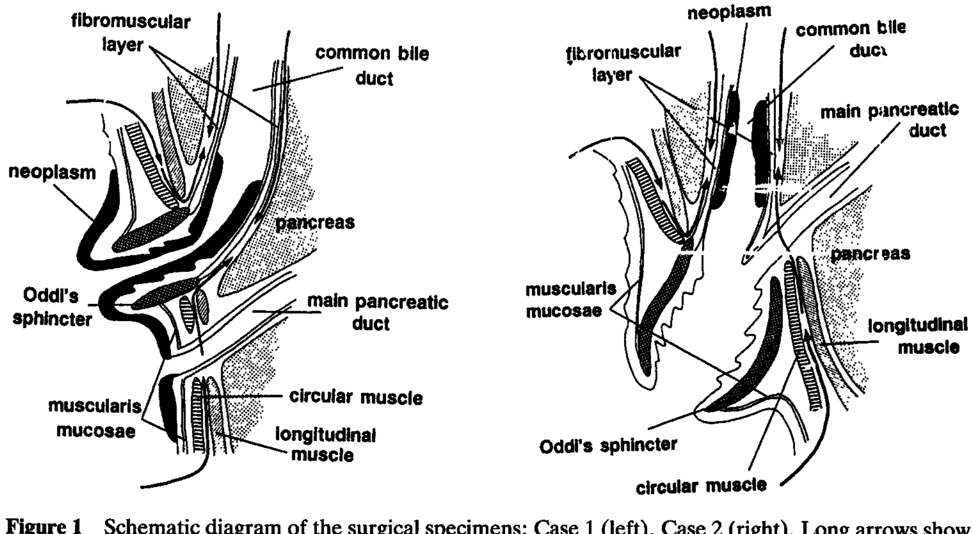

**Figure 1** Schematic diagram of the surgical specimens: Case 1 (left), Case 2 (right). Long arrows show the surgical plane of dissection.

without invasion to the deeper layers (muscularis mucosae, sphincter of Oddi, fibromuscular layer of the bile duct (Figure 2).

*Case 2*

A 70-year-old woman underwent cholecystectomy and choledochotomy with T tube drainage. Gall stones were confined to the gallbladder, but operative cholangiography suggested a polypoid lesion in the terminal bile duct. Liver function tests had nearly returned to normal by the time she arrived in our unit. T tube cholangiography and endoscopic retrograde cholangiography showed a 5 mm filling defect proximal to the confluence of the main pancreatic duct (Figure 3). Cholangioscopy through the T tube tract showed a polypoid lesion in the terminal bile duct; endoscopic biopsies demonstrated a neoplastic lesion without evidence of stromal invasion. At operation the neoplastic lesion was thought to be located mainly in the terminal bile duct, so the papilla and common bile duct were resected in continuity in essentially the same manner as above (Figure 3). The pancreatic duct formed a common channel with the bile duct in this case, and it was resutured to the duodenal mucosa. The patient remains well with no evidence of recurrence 30 months postoperatively.

Pathological study demonstrated a flatly elevated tumour (6 × 4 mm) located in the terminal common bile duct 12 mm proximal to the duodenal lumen. Histologically it was a well-differentiated tubular adenocarcinoma confined to the mucosa.

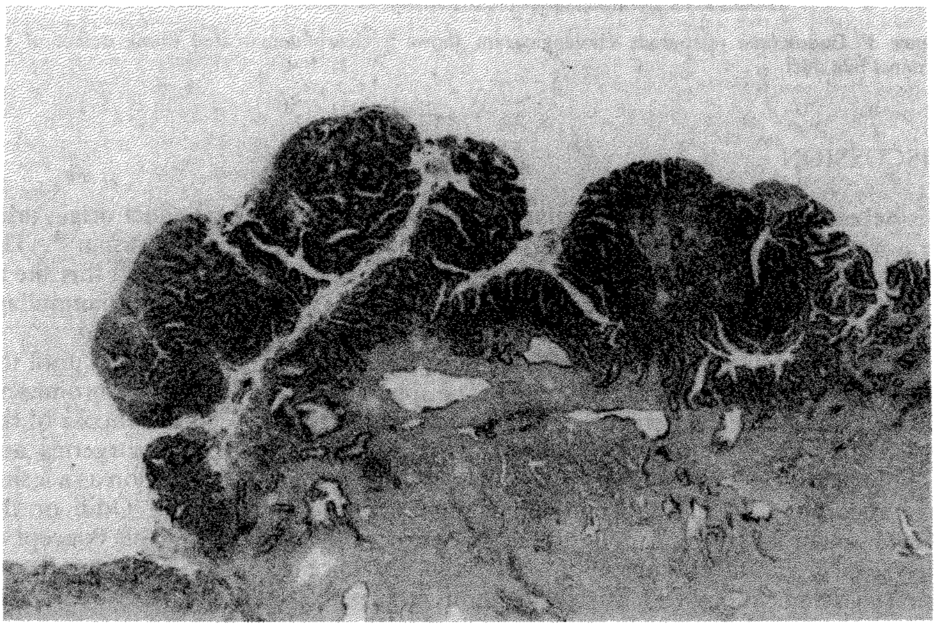

**Figure 2** Microscopic picture of the papilla of the Vater of the surgical specimen (Case 1) showing well-differentiated papillotubular adenocarcinoma replacing the papilla of Vater and the terminal bile duct, without invasion to Oddi's sphincter. (Hematoxylin and eosin stain; original magnification ×40).

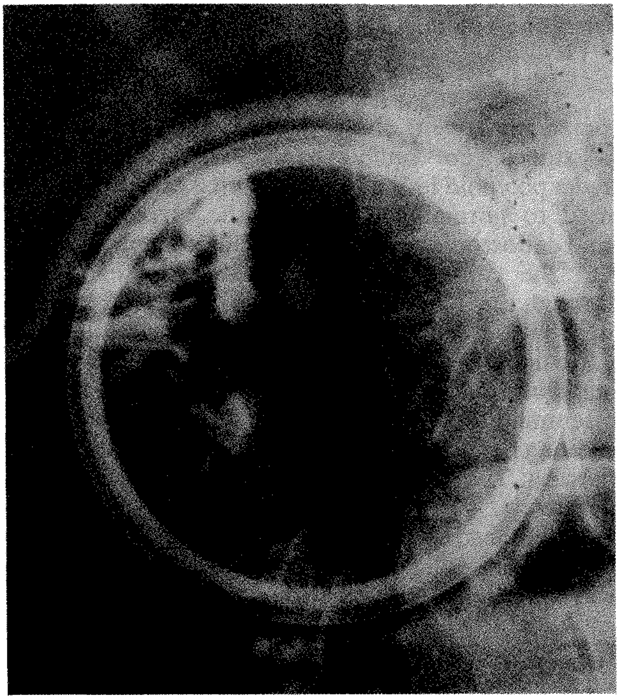

**Figure 3** Endoscopic retrograde cholangiogram shows a clearly-demarcated filling defect of the terminal bile duct.

### DISCUSSION

It is debatable whether periampullary cancers of duodenal or bile duct origin arise de novo from normal mucosa or develop from a pre-existing adenoma[2–6]. The concept of an adenoma-carcinoma sequence is not as well established at this site as it is for colonic neoplasms. For practical purposes it is best to treat periampullary lesions on the assumption that they are potential carcinomas.

With carcinoma of the stomach and colon, metastases are rarely found until the muscularis mucosae has been breached. The sphincter of Oddi and the fibromuscular layer of the bile duct corresponds anatomically to the muscularis mucosa of the duodenum. Using the analogy of early gastric cancer, Japanese surgeons and pathologists have tried to define early cancer of the periampullary region as a lesion that does not infiltrate the muscularis mucosae, the sphincter of Oddi or the fibromuscular layer[3,9,10]. Among the small number of cases studied, this concept of early cancer has been substantiated by the abscence of lymph-node or distant metastasis and by long-term survival without recurrence. Until now, however, most patients with early cancer have been treated with pancreatoduodenectomy, since tumour extension could not be precisely evaluated.

Local excision and reconstruction of the ampulla was described by Halsted in 1899[11]. In 1951, Miller *et al.* reported 13 patients with cancer of the papilla who underwent transduodenal excision with or without transplantation of the duct(s); there was a high operative mortality rate of 46%[12]. Six of 7 patients who survived died of recurrent cancer within 26 months. This experience suggested that local excision might be inappropriate for cancer of the papilla of Vater except for high-risk patients. Later Archie *et al.* reported a case of polypoid adenoma of the ampulla, which was resected locally followed by double sphincteroplasty[13].

Krukowski and colleagues have recently described in detail the procedure performed for three patients with tubulovillous adenoma. Submucosal resection of the periampullary tumours was extended with intraduodenal excision of the distal bile and pancreatic ducts[14]. Our operation differs by including the papilla and the whole length of the common bile duct in the resection and by dissecting in the plane between the inner circular muscle and the outer longitudinal muscle of the duodenum (below) and outside the muscle of the ampulla and the fibromuscular layer of the bile duct (above). It is one option for a periampullary tumour with uncertain mucosal spread, especially a lesion arising in the terminal bile duct and extending to the papilla. Since superficial biopsies are unreliable, repeated frozen-section analysis will be needed to rule out invasive carcinoma as the dissection proceeds.

It is well known that villous adenoma of the periampullary region has the same malignant potential as villous adenoma of the large intestine[15]. Among Gray's 38 cases of villous tumours of the ampulla[5] (the largest series to-date), there were 21 patients with benign adenoma, 3 with carcinoma *in situ*, and 14 with carcinoma. Papillocholedochectomy might have been indicated for more than half of this group, namely those with adenoma and carcinoma *in situ*.

### *References*

1. Perzin, K.H. and Bridge, M.F. (1981) Adenomas of the small intestine. A clinicopathologic review of 51 cases and a study of their relationship to carcinoma. *Cancer*, **48**, 799–819
2. Baczako, K., Buchler, M., Berger, H.G., Kirkpatrick, J. and Haferkamp, O. (1985) Morphogenesis and possible precursor lesions of invasive carcinoma of the papilla of Vater; Epithelial dysplasia and adenoma. *Human Pathology*, **16**, 305–310
3. Yamaguchi, K. and Enjouji, M. (1987) Carcinoma of the ampulla of Vater; A clinicopathological study and pathologic staging of 109 cases of carcinoma and 5 cases of adenoma. *Cancer*, **59**, 506–515
4. Sekoguchi, T., Nakamura, K., Iwasa, M., Kitamura, J., Katumine, Y. *et al.* (1988) The significance of adenoma in the histogenesis of cancer of the papilla of Vater. *Tan to Sui*, **9**, 81–89 (in Japanese)
5. Kozuka, S., Tsubone, M. and Hachisuka, K. (1984) Evolution of carcinoma in the extrahepatic bile ducts. *Cancer*, **54**, 65–74
6. Kozuka, S., Tsubone, M., Yamaguchi, A. and Hachisuka, K. (1981) Adenomatous residue in cancerous papilla of Vater. *Gut*, 1031–1034
7. Ikejiri, T., Ushijima, K., Kobayashi, M., Yamaguchi, K. and Inokuchi, K. (1977) Early carcinoma in the periampullary region: Report of two cases. *Jap. J. Surg.*, **7**, 28–34
8. Gray, G. and Browder, W. (1989) Villous tumors of the ampulla of Vater: Local resection versus pancreatoduodenectomy. *Southern Med. Journal<ie>*, **82**, 917–920
9. Hanyuu, F., Imaizumi, T., Nakamura, K. and Yoshikawa, T. (1984) The concept of early cancer of the papilla of Vater. *Tan to Sui*, **5**, 847–852 (in Japanese)
10. Hanyuu, F., Eguchi, R., Nakamura, K., Imaizumi, T., Yoshikawa, T., Ohashi, M., Suzuki, M. and Miura, O. (1987) Clinicopathological study of long-term survivors with bile duct cancer. *Tan to Sui*, **8**, 1197–1203 (in Japanese)
11. referred from (1). Halstead, W.S.: Contributions to the surgery of the bile passages especially of the common bile duct. *Boston Med. Surg.*, (1899), **141**, 645–654
12. Miller, E.M., Dockerty, M.B., Wollaeger, E.E. and Waugh, J.M. (1951) Carcinoma in the region of the papilla of Vater. *Surg. Gynecol. Obstet.*, **92**, 172–182

13. Archie, J.P., Jr., Murray, H.M., Jr. (1978) Benign polypoid adenoma of the ampulla of Vater. *Arch. Surg.*, **113**, 180–181
14. Krukowski, Z.H., Ewen, S.W.B., Davidson, A.I. and Matheson, N.A. (1988) Operative management of tubulovillous neoplasms of the duodenum and ampulla, *Br. J. Surg.*, **75**, 150–153
15. Ryan, D.P., Schapiro, R.H. and Warshaw, A.L. (1986) Villous tumors of the duodenum. *Ann. Surg.*, **203**, 301–306

*(Accepted by S. Bengmark 31 March 1992)*

### INVITED COMMENTARY

This paper by Dr Kasahara and his colleagues focuses on a current clinical dilemma: how best to treat ampullary neoplasms that appear benign or of low grade malignancy, bearing in mind that preoperative and even intraoperative biopsy can miss foci of invasive carcinoma. Tubulovillous and villous adenomas of the papilla or periampullary duodenal mucosa have both a high incidence of associated malignancy when examined *in toto* and a high rate of future malignant transformation if left *in situ*[1,2]. By analogy with the colon, adequate local excision should cure a lesion that does not show invasive malignancy, while invasive lesions can often be cured by pancreatoduodenectomy (with a five year survival rate of up to fifty percent)[3,4]. If all patients with ampullary neoplasms are subjected to a pancreatoduodenectomy, some with "benign" lesions may be undergoing a more major procedure than is necessary for cure. By contrast, if all those without preoperative or intraoperative evidence of invasive malignancy are treated by means of local excision, those with unsuspected invasive cancer will receive an inadequate operation and are at risk of recurrence. To further complicate matters, many patients with ampullary cancer are elderly and deemed unfit for radical resection, although Kairaluoma and co-workers report low morbidity and mortality rates for pancreatoduodenal resection in patients over the age of seventy years[5]. For the aged and infirm, local resection or even endoscopic sphincterotomy through the tumour (with or without stenting) are palliative options[6,7].

The authors rightly do not attempt to resolve this debate on the basis of their two case reports. Rather, they introduce another treatment option (papillocholedochectomy) that may be useful in at least two situations. If local resection of an ampullary tumour is undertaken and frozen section examination shows extension to the bile duct margin, this technique may allow proximal clearance to be attained. Alternatively, after completing an adequate local resection of the ampulla the surgeon might be concerned that anastomosis of the terminal bile duct to the duodenum would create undue tension, in which case the authors' technique may allow a safer reanastomosis. The pancreatic duct remains to be dealt with in the usual fashion.

Several recent trends may alter the debate on treatment of these difficult lesions. Although Knox and Kingston found a better five-year survival with local excision of ampullary carcinoma than with radical resection, their operative mortality rate for pancreatoduodenectomy (30 percent) vitiated the comparison[6]. Fortunately the incidence of complications and death following resection of the pancreatic head has sharply decreased in recent years[8]. Morever, Ryan *et al.* have described tumour recurrence following local excision of a lesion that was benign on complete pathological examination[1]. The papillo-choledochectomy described by Kasahara *et al.* involves a duodenal suture line, a pancreatico-enteric anastomosis, creation of a Roux limb, excision of the common bile duct, a bilio-enteric anastomosis and possibly a cholecystectomy. It remains to be seen where this procedure will come to lie on the diminishing spectrum of morbidity and mortality between local excision and radical resection. It seems probable that pancreatoduodenectomy in expert hands will emerge as the definitive treatment for most ampullary neoplasms.

R. Howerton
R.C.N. Williamson
Directorate of Surgery
Hammersmith Hospital
DuCane Road
London W12 0NN, UK

### *References*

1. Ryan, D.P., Schapiro, R.H. and Warshaw, A.L. (1986) Villous tumors of the duodenum. *Ann. Surg.*, **203**, 301–306
2. Perzin, K.H. and Bridge, M.F. (1981) Adenomas of the small intestine. A clinicopathological review of 51 cases and a study of their relationship to carcinoma. *Cancer*, **48**, 799–819
3. Komorowski, R.A. and Cohen, E.D. (1981) Villous tumours of the duodenum: a clinicopathologic study. *Cancer*, **47**, 1377–1386
4. Neoptolemos, J.P., Talbot, I.C., Carr-Locke, D.L. *et al.* (1987) Treatment and outcome in 52 consecutive cases of ampullary carcinoma. *Br. J. Surg.*, **74**, 957–961
5. Kairaluoma, M.I., Kiuiniemi, H. and Stahlberg, M. (1987) Pancreatic resection for carcinoma of the pancreas and periampullary region in patients over 70 years of Age. *Br. J. Surg.*, **74**, 116–118
6. Knox, R.A. and Kingston, R.D. (1986) Carcinoma of the Ampulla of Vater. *Br. J. Surg.*, **73**, 72–73
7. Bickerstaff, K.I., Berry, A.R., Chapman, R.W. and Britton, B.J. (1990) Endoscopic sphincterotomy for the palliation of ampullary carcinoma. *Br. J. Surg.*, **77**, 160–162
8. Trede, M., Schwall, G., Saeger, H.D. (1990) Survival after pancreatoduodenectomy: 118 consecutive resections without operative mortality. *Ann. Surg.*, **211**, 447–458

